# Piezo Ion Channels and Their Association With Haptic Technology Use: A Narrative Review

**DOI:** 10.7759/cureus.77433

**Published:** 2025-01-14

**Authors:** Jeffrey Gudin, Mark Sakr, Janet Fason, Peter Hurwitz

**Affiliations:** 1 Pain Medicine, University of Miami, Miami, USA; 2 Sports Medicine, University of Arizona, Tucson, USA; 3 Family Medicine, Stein Medical, Tyrone, USA; 4 Research, Clarity Science LLC, Narragansett, USA

**Keywords:** all neurology, connectomics, haptic exposure, haptic vibrotactile trigger technology, ion channels, multi-modality pain management, piezo, sensory perception, tactile perception

## Abstract

The recent identification of Piezo ion channels demonstrating a mechano-sensitive impact on neurons revealed distinct Piezo-1 and 2 types. While Piezo-1 predominates in neurons linked to non-sensory stimulation, such as pressure in blood vessels, Piezo-2 predominates in neurons linked to sensory stimulation, such as touch. Piezo-1 and 2 have a major bidirectional impact on transient receptor potential (TRP) ion channels, and TRPs also impact neurotransmitter release. Particularly existent in dorsal root ganglion (DRG) neurons, which are located in nerve endings, Piezo-2 is a key DRG activator. Subsequent Piezo findings have been vital to recent medical haptic technology developments, in tandem with breakthroughs in the emerging neurology subfield of *connectomics* plus AI developments. Included in this review are a historical Piezo overview, the interrelationship of Piezo channels with TRPs, inclusive of TRPV1/TRPV8, the impact on medical and rehab haptic technology, a focus on haptic technology use in stroke survivor rehab inclusive of pain mitigation, and the development of a haptic technology patch aimed at alleviating pain and/or anxiety. Neurogenic pain resulting from hyperalgesia/allodynia in stroke survivors is a potential target for drugs and haptics aimed at pain reduction; patients experiencing neuropathic or psychosomatic pain are other prime targets. Increased Piezo knowledge may promote more precisely targeted haptic therapeutic developments.

## Introduction and background

Ion channels control the transmission of neural impulses in the peripheral and central nervous systems and are responsible for the biology of the senses. Specific ion channels, such as the transient receptor potential (TRP) channel, respond to various stimuli, such as temperature, touch, and vibration, whose responses are conveyed from the skin to the brain [1]. Other ion channels, such as Piezo-1 and 2, are mechano-transducing (mechano-sensitive) and open in response to mechanical stimuli [2,3]. Mechano-transduction allows living organisms to receive and respond to physical signals from the external and internal environments; Piezo channel research has revealed it to be among the largest mechano-sensitive ion channels [2] than other ion channels (e.g., potassium channels), which may also be large in size but function differently. The brain's ion channels also act on neurotransmitter (e.g., dopamine) release and other secreted biochemicals [4].

Haptic technology creates a sense of touch using vibration, motion, and other forces and has been gaining popularity in medical arenas since the 1980s-1990s. One example in medical education is the use of surgical simulators that enable tactile feedback to surgeons-in-training [5]. Haptic technology has also been incorporated into diverse stroke and traumatic brain injury (TBI) rehab devices, especially in tandem with virtual reality (VR) technology. Furthermore, the "cutting-edge" use of medically focused haptic technology has expanded to include pain, sleep, and anxiety relief [6-8]. Ongoing research will support these advances in haptic technologies and their incorporation into various medical applications.

Historical overview of Piezo ion channel knowledge base

First discovered by Ardem Patapoutian and his team in 2010 (resulting in the receipt of a Nobel Prize in 2021), Piezo-1 and 2 were identified as ion channels in the plasma membrane of neuronal cells that can be activated in response to stimuli such as temperature, chemicals, and mechanical force [9]. Moreover, Piezo-1 and 2 together were discovered to be the most major non-selective mechano-sensitive ion cation channels in the cell membrane [10] (cation channels are protein pores embedded in a neuron's membrane that allow passage into and through it of positively charged ions). Subsequent to these discoveries, various investigations to understand the comprehensive structure of Piezo ion channels commenced, including how they function to generate diverse physiological responses [10]. In contrast to Piezo-1, which senses mechanical forces such as pressure in non-sensory cells (e.g., lung, bladder, and skin), Piezo-2 is primarily found in sensory neurons. Notably, for its impact on recent haptic technology developments, Piezo-2 is especially found in dorsal root ganglion (DRG) neurons and also Merkel cells (found right below the skin's epidermis and interacting with nerve endings) [10]. Piezo and temperature-sensing (TRP) channels interact to modulate biological signaling in all tissues. Piezo channel volume and distribution may shift in the face of injury. These channels play a role in regulating neurogenesis (axon growth) in both the developing and injured brain. This has led to recent interest in Piezo modulation for conditions such as TBI and stroke [11,12].

**Figure 1:**
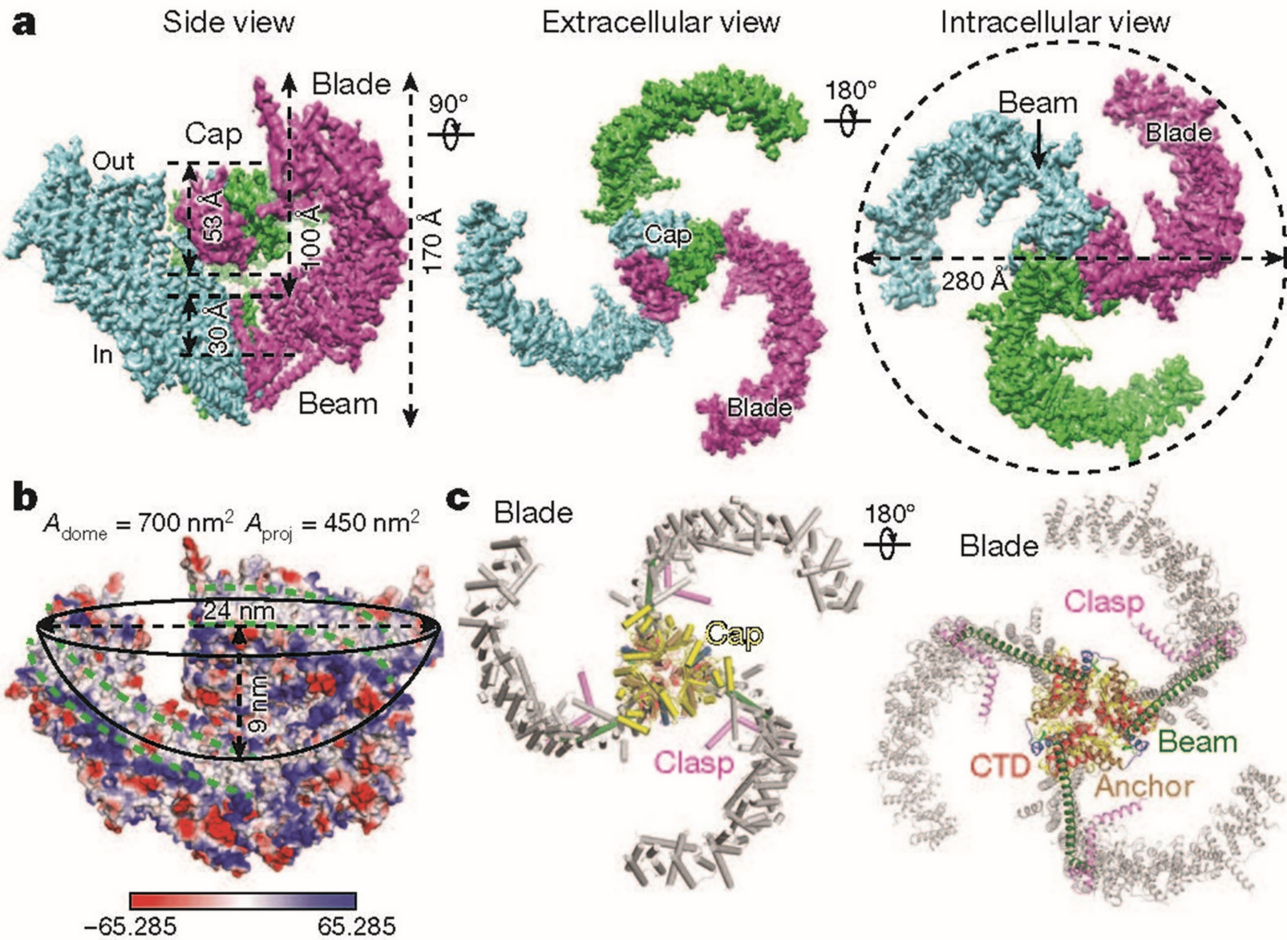
The homotrimeric structure of Piezo-2. Reprinted with permission from Wang et al. [15]

Investigations of Piezo ion channels have revealed that they are trimers and shaped like a three-bladed propeller (Figure 1) [13], and Piezo proteins have a large predicted size of approximately 2,500 amino acids [14]. However, Piezo-1 and Piezo-2 share only 42% of amino acid sequence homology [14]. In terms of kinetics, Piezo ion channels can be modeled into three states: open, closed, and inactive [14]. Among its myriad impacts throughout the body, Piezo-1 is involved in modulating cardiac homeostasis, vascular tone, and blood pressure, while Piezo-2 is crucial to both touch sensation and proprioception [14]. The growing base of knowledge pertaining to ion channel functions, including Piezo-1 and 2, has been instrumental to medical haptic technology developments. In addition, it may contribute to pharmacological research and development (R&D), targeting modulators for specific neurodegenerative disorders.

## Review

Relationship of Piezo-2 to DRG neurons

Piezo-2 is expressed by all DRG neurons and plays a key role in the activity of these neurons [3]. DRG neurons are bidirectional sensory neurons that detect and transmit information about peripheral stimuli to the central nervous system (CNS). Consequently, DRG neurons have been an intervention target for the modulation of pain. One study in 2021 showed that increased Piezo-2 expression was linked to increased pain, and the presence of Piezo-2 in DRG neurons may underpin the effectiveness of certain haptic therapies and certainly warrants further investigation [3].

Relationship of Piezo ion channels to TRP ion channels

Elucidating the full scope of the relationship between Piezo and TRP ion channels has the potential to improve pharmaceutical and other medical interventional R&D, as well as medical haptic technology R&D. Piezo ion channels and TRP ion channels are both considered to be mechano-sensitive on a cellular level. These two categories of ion channels can exert an impact on each other. These mechano-sensitive receptors constitute a "family" of ion channels that respond in distinct ways to physical force or plasma membrane deformations and represent the first step toward the conversion of mechanical stimuli to a biochemical or electrical response [16]. In addition, both Piezo and TRP ion channels regulate and affect numerous cellular processes, such as the inflammatory responses of immune cells, cell migration, and cell birth/death. Thus, a complex relationship between Piezo and TRP ion channels exists, prompting an effect that can generate a physiological impact. There are six different families of TRPs with specific roles, and these are classified as TRP C (canonical), TRP V (vanilloid), TRP M (melastatin), TRP A (ankyrin), TRP P (polycystin), and TRP ML (mucolipin) [17]. Many are familiar with TRP V (capsaicin-modulated) and TRP M (menthol-modulated) receptor actions. In this regard, there are still many unanswered questions concerning these channels. Among them, the mechanisms of temperature activation, such as TRP V1 by heat or TRPM8 by cold, and the mechanism of mechanosensing by Piezos remain among the most pressing unanswered questions. Recognition of the Nobel Prize not only honors the seminal discoveries of these channels but also stimulates excitement among structural biologists to continue pursuing these burning questions.

Interrelationship of Piezo ion channels and TRP V1 plus TRP M8

While TRP ion channels are overall thermal-sensitive, Piezo ion channels are overall pressure-sensitive [18]. As noted above, due to the significant interplay between TRP and Piezo channels, each can affect the functioning of the other. Notably, David Julius was co-awarded in 2021, along with Ardem Patapoutian, the Nobel Prize in Physiology or Medicine. Julius identified the TRP V1 ion channel as a heat-activated nociceptor in the peripheral nervous terminus, which is the part of the mature nociceptor where noxious stimuli are detected and transduced into electrical energy [9]. Both Patapoutian and Julius independently identified TRP M8 as the cold-activated nociceptor [9].

Following Julius' discovery, researchers found that TRP V1 ion channels inhibited the activity of Piezo-1 and Piezo-2 channels; this occurred through the depletion of neuronal membrane phosphoinositides [19]. Meanwhile, TRP M8, similar to others in the TRP melastatin subfamily, is activated by cool temperatures and certain cooling compounds (e.g., menthol and eucalyptol).

Notably, both TRP V1 and TRP M8 can impact pain perception, as can Piezo ion channels. Therefore, additional research on the impacts of Piezo and TRP is warranted to further the development of new drugs or devices that more effectively control the nociceptive responses to pain.

Interrelationship of TRP ion channels on neurotransmitters affecting anxiety and pain perception

Neurotransmitters are brain biochemicals that amplify, transmit, and convert signals in cells and play a vital role in information transmission throughout the CNS and peripheral nervous system (PNS) [20]. Some examples, such as serotonin, dopamine, GABA, and norepinephrine, are particularly recognized as affecting emotions, anxiety levels, and pain perception [20,21]. Serotonin, or 5-HT, is a neuromodulator that can stimulate the TRP V1 channel in the periphery; in turn, TRP V1 can influence dopamine release. Consequently, TRP V1 is partially regulated by neurotransmitters, including those vital to the modulation and perception of signals that influence emotions, anxiety, and/or pain perception [22].

Focus on dopamine as a neurotransmitter affecting anxiety and pain

There are five dopamine receptors in humans, and a primary role of the brain's dopamine-1 (D1) receptor is stimulating the production of cyclic adenosine monophosphate (cAMP) when activated by dopamine release to enable neuron excitability; in turn, this excitability is linked to age-related and neurodegenerative cognition changes. In contrast, the dopamine-2 (D2) receptor inhibits the production of cAMP, with an inhibitory effect on neuron excitability. Therefore, the D1 and D2 receptors complement each other while performing opposing roles. Piezo channels have been located on microglia in the CNS and may be targets for neurodegenerative diseases such as Alzheimer's disease (AD). Although beyond the scope of this review, microglia are endogenous immune cells that maintain brain homeostasis. In AD, toxic amyloid beta (Aβ) accumulates in the brain and forms stiff plaques. Research has shown that Piezo-1 is present and functional in microglia and may be a target for interrupting the pathological processes associated with certain CNS conditions [23].

Tactile stimulation and the release of dopamine or serotonin

Both dopamine and serotonin release are increased during pleasurable activities and tactile stimulation [24,25]. The use of haptic technology (including both portable and wearable devices) aimed at tactile stimulation is increasingly being embraced, focusing on three types of mechanisms. These mechanisms can be categorized as force-based haptic feedback interfaces, thermal-based haptic interfaces, and nerve-stimulation haptic feedback interfaces [26]. Force-based haptics uses force feedback to the user's hands to simulate tactile sensations; thermal interfaces modify the temperature felt on the skin; and nerve-stimulation interfaces with the Piezo-2 ion channels to stimulate the DRG neurons to transmit neural signals to the brain.

Connectomics and its potential impact on sensory-based haptic technology

Breakthroughs in neuroscience have led to the emergence of a new field of neuroscience: Connectomics [27]. This neuroscientific field is concerned with the comprehensive mapping of the connections between neurons in the brain. This mapping is simultaneously occurring on the three mapping levels of microscale, mesoscale, and macroscale [28]. A study of connectome architecture focused on sensory-motor integration revealed that signal spreading occurs faster from nodes associated with sensory transduction (sensors) to nodes associated with motor output (effectors) [29]. Even more recently, researchers, in an effort to better understand the way sensory stimuli elicit signaling events within structural connectivity networks, which then manifest as patterned neural activity, have developed an artificial intelligence (AI)-driven connectome-based reservoir computing generator (conn2res) that may expedite this entire mapping process [30].

As Sadaghiani et al. concluded in 2022, electrophysiology connectomics may be poised to inform testable mechanistic models of information in hierarchical brain networks [31]. While similar in some ways to the mapping of the human genome, this connectome-mapping process is more complex. For this reason, developments in AI have been crucial to this mapping progress.

Meanwhile, novel developments pertaining to memristors, which are nonvolatile electronic components that regulate the flow of electrical current in a circuit and "remember" the amount of the charge that has passed through it, also need to be recognized. Recent advances in nanoelectronics have made possible the fabrication of devices that exhibit memristor-like characteristics; it was posited by a research team more than five years ago that memristors are the fourth fundamental circuit element, besides the other three known elements of resistor, inductor, and capacitor [32]. Along with the expansion of the connectomics knowledge base, the development of novel memristors increases the potential for the future of sensory-based haptic technology treatments.

Haptic technology utilization to target specific disorders

Haptic stimulation is currently being used as a noninvasive treatment for adults living with diverse neurodegenerative disorders. For adults post-stroke or TBI (who often have resulting sensory deficits), haptic stimulation is used as a rehab approach to promote sensory retraining [33]. For people with Parkinson's disease, vibrotactile (VTT) stimulation at gamma frequency has been shown to lessen cognitive decline and improve motor function [34], and haptic stimulation has also been found to attenuate AD symptoms [35]. In addition, the findings of a recent clinical trial of a haptic VTT patch in adults with symptoms of emotional stress/anxiety showed reduced anxiety and other improved mental health variables [36].

Pain has been a particular focus as a potential for therapeutic VTT haptic sensory stimulation, and numerous studies have shown that mechanical stimuli generate a Piezo channel response that can lessen pain [37]. Vibrating motors were used on the fingers of the remaining hand in research subjects with an amputated hand to reduce the "phantom pain" in the amputated hand [38]. In a different study, a haptic patch was worn and utilized by research subjects diagnosed with pain [39]. The findings pertaining to the use of both of these modalities revealed an achieved reduction in self-perceived pain.

Mental health disorders have been another focus as a potential for therapeutic VTT haptic sensory stimulation [37,40], as well as VR devices with vibrotactile platforms [41]. These disorders have ranged from anxiety and phobias to schizophrenia [41]. Since the perception of pain can be distorted (either abnormally increased or decreased) in some adults diagnosed with psychiatric disorders [42], chronic pain as a VTT target can be investigated as a distinct therapeutic target (in and of itself) or included as a possible future therapy aimed at persons living with a mental health disorder resulting in pain perception distortions. Moreover, since chronic depression has been linked to increased pain perception [43], the use of VTT haptic sensory stimulation for the treatment of chronic depression in these people may help alleviate their pain, resulting in lessened depression.

Haptic technology utilization: focus on stroke rehabilitation use

Stroke is the fifth-leading cause of death in the US [44], and stroke prevalence in the US increased from 2011-2013 to 2020-2022 by 14.6% and 15.7% among adults aged 18-44 and 45-65, respectively [45]. Stroke is also a leading cause of disability both in the US and globally. Sensory impairment is common in stroke survivors and can be featured by reduced ability to feel touch or differentiate between heat and cold, as well as numbness and tingling. The cause is frequently due to primary or secondary injury to the thalamus or the neural pathways connecting the thalamus to the parietal lobe(s) [46].

Problematically, thalamic pain syndrome (TPS) is a potential consequence of thalamic injury, and TPS is typically characterized by hyperalgesia (increased sensitivity to pain) and/or allodynia (misperception of pain from nonpainful stimuli contact) [47]. Stroke survivors with TPS are far less likely to participate fully in prescribed stroke rehab exercises to regain lost motor skills and also cognitive skills [47], and physical therapy after a stroke is most effective when begun within the first three months post-stroke [48]. Thus, broader therapeutic options for TPS are needed besides medications and invasive treatments. This is necessary because not all patients with TPS can tolerate or respond to the current available therapies.

Hand and finger dexterity impairments are common post-stroke, and haptic robotic devices are utilized as neuro-rehabilitation tools aimed at the hand and fingers [49,50]. Haptic feedback patches applied to the trunk or shoulders have also been used to improve balance (and lessen trunk sway) [51]. In addition, VR gaming incorporating haptic feedback has been used to improve cognitive functioning [52].

Haptic technology use in substance abuse treatment

At least 60% of people treated for a substance use disorder (SUD) relapse within the first year of treatment, and that relapse is often preceded by an overwhelming craving for the substance (e.g., heroin) [53]. Chronic substance abuse alters various neurotransmitter systems [54], including dopamine release. Furthermore, chronic opioid drug abuse is associated with reductions in dopamine release; D2 receptors in the brain's striatum are associated with reduced activity of the orbitofrontal cortex (region involved with motivation and compulsivity) along with the cingulate gyrus (region involved with inhibitory control and impulsivity) [55], which may exacerbate the cycle of addiction and relapse.

VR treatments incorporating haptic technology have been used to reduce the cravings leading to relapse [56]. It has also been used in treating substance abusers due to its therapeutic effects on depression and anxiety, which are common co-disorders; both depression and anxiety can promote substance abuse relapse occurrences [57]. Notably, sensory feedback is one of the key elements of VR therapeutics incorporating haptic technology aimed at substance abuse treatment [56].

## Conclusions

Since its discovery in 2010, research investigations on the Piezo ion channel's impact on the brain and nervous system have increased, as have studies focused on TRP V1 and TRP M8. The ability to detect and respond to changes in the extracellular environment is critical for all neuronal cells, so determining the sensory roles of specific ion channels is an important scientific breakthrough with huge potential medical intervention implications.

VTT haptic technology is grounded in an understanding that TRP ion channels stimulate the brain's sensory (and other) responses to peripheral nerve stimuli, such as those that exist in the skin. Touch perceptions are bidirectional, and VTT-induced sensations on the skin can promote brain neurotransmitter reactivity, brain "neuroplasticity," and improved cognitive functioning. Overall, VTT haptic technology in the form of a noninvasive patch is especially promising for self-use by people living with various physical and/or mental health disorders. Further investigation is warranted to determine the best types of haptic stimulation, locations for those stimuli, and other to-be-determined markers that will affect the potential success of topical Piezo-modulating haptic therapies. Rapid breakthroughs in the emerging neuroscience field of connectomics, combined with the current developments in AI aimed at the medical arena, suggest that the future is bright for developing diverse, noninvasive, drug-free technologies, such as VTT haptics, that can be used to improve the symptoms of patients living with diverse disorders. Recent research with haptics has shown positive outcomes in patients experiencing chronic pain, sleep disorders, anxiety, and other disorders. This will be especially useful for disorders where the currently available treatments (e.g., pharmacological, surgical procedures, acupuncture, and other complementary therapies) are suboptimal, may have significant side effects, and provide only limited relief.
